# Reply to Dr Hooda Z

**DOI:** 10.1093/icvts/ivaf079

**Published:** 2025-04-11

**Authors:** Asato Hashinokuchi, Takaki Akamine, Tomoyoshi Takenaka, Tomoharu Yoshizumi

**Affiliations:** Department of Surgery and Science, Graduate School of Medical Sciences, Kyushu University, Fukuoka, Japan; Department of Surgery and Science, Graduate School of Medical Sciences, Kyushu University, Fukuoka, Japan; Department of Surgery and Science, Graduate School of Medical Sciences, Kyushu University, Fukuoka, Japan; Department of Surgery and Science, Graduate School of Medical Sciences, Kyushu University, Fukuoka, Japan

**Keywords:** Spread through air spaces, Non-small cell lung cancer, STAS distance

Dear Editor,

We would like to thank Dr Hooda and Antonoff for their valuable comments on our study regarding the clinical significance of the distance of spread through air spaces (STAS) in non-small-cell lung cancer (NSCLC) [1]. We agree with Dr Hooda that STAS is useful for predicting poor prognosis in patients with NSCLC and that improving diagnostic and therapeutic modalities for STAS-positive NSCLC will contribute to better outcomes.

As Dr Hooda noted regarding the definition of STAS distance, 2 previous reports used the number of alveoli from the main tumour, a method different from that used in our study. However, these studies did not identify significant prognostic differences based on STAS distance [2]. In contrast, we evaluated the maximum spread distance from the tumour edge to the farthest STAS in each case. This definition of STAS distance aligns with 4 previous reports, and in 3 of the 5 studies, including our analysis, STAS distance was a prognostic factor in patients with STAS-positive NSCLC [2]. Thus, we believe that measuring the distance between the STAS and the tumour edge is a reproducible method.

A recent clinical trial showed that segmentectomy can provide survival outcomes comparable to lobectomy for small peripheral NSCLC, suggesting that segmentectomy may become the standard surgical procedure [3]. However, sublobar resection, including partial resection and segmentectomy, has been associated with a higher local recurrence rate than lobectomy in patients with STAS-positive NSCLC [4]. In contrast, several studies have suggested that outcomes after segmentectomy may be acceptable and comparable to those after lobectomy in patients with STAS-positive NSCLC [5]. Thus, the optimal surgical procedure for patients with STAS-positive NSCLC remains controversial.

The presence of STAS did not aid in determining the surgical procedure, as STAS evaluation was performed postoperatively. Notably, as Dr Hooda mentioned, intraoperative evaluation of STAS using frozen sections exhibits high specificity and may help in selecting an optimal surgical procedure. Furthermore, Tao *et al.* [6] reported that an artificial intelligence model using computed tomography was useful for preoperative prediction of STAS. In this study, the area under the receiver operating characteristic curve was 0.93 in the training cohort and 0.80 in the validation cohort. These findings indicate ongoing efforts to predict STAS before a final pathological diagnosis. We hope that these results will have clinical applications.

Our study on STAS distance highlights the prognostic significance of STAS in NSCLC. Furthermore, as outlined above, assessing STAS may be useful in determining surgical strategy.

## Data Availability

The data underlying this article will be shared upon reasonable request from the corresponding authors.
